# Clinical experience with alteplase in the management of intracardiac and major cardiac vessels thrombosis in pediatrics: a case series

**DOI:** 10.4103/0256-4947.62840

**Published:** 2010

**Authors:** Abdulrazaq S. Al-Jazairi, Roaa A. Al-Gain, Zead R. Bulbul, Antoine J. Cherfan

**Affiliations:** a From the Division of Pharmacy Services, King Abdulaziz Medical City, Riyadh, Saudi Arabia; b From the King Faisal Heart Institute King Faisal Specialist Hospital and Research Centre, King Abdulaziz Medical City, Riyadh, Saudi Arabia; c From the Critical Care Pharmacy Department, King Abdulaziz Medical City, Riyadh, Saudi Arabia

## Abstract

**BACKGROUND AND OBJECTIVES:**

Experience with alteplase in pediatric patients is limited and recommendations are extrapolated from adult data. Comprehensive guidelines on the management of thromboembolic events in this group are lacking. We assessed the efficacy and safety of alteplase (recombinant tissue plasminogen activator) in the management of intracardiac and major cardiac vessel thrombosis in pediatric patients.

**METHODS:**

All pediatric patients, 14 years of age and younger, with intracardiac or major cardiac vessel thrombus who were treated with alteplase from 1997 to 2004 at our tertiary care institute were identified through the pharmacy database. Patient data were retrospectively evaluated for the efficacy and safety of altepase.

**RESULTS:**

Five cases were eligible out of nineteen who received alteplase. Patient ages ranged from 40 days to 13 years. The initial dose of alteplase ranged from 0.3 to 0.6 mg/kg followed by a continuous infusion in three patients with a dosage range between 0.05 and 0.5 mg/kg/hr, while intermittent infusion was used in the other two patients. The duration of therapy ranged from 2 to 4 days. By the end of the treatment, two patients had complete resolution of thrombus and one had partial resolution. Two patients failed to respond and had “old” thrombus. Major bleeding events were reported in three patients. The rest had minor bleeding events.

**CONCLUSION:**

Alteplase may effectively dissolve intracardiac thrombi, particularly when freshly formed. Continuous infusion for a long duration appears to be associated with an increased risk of major bleeding. Optimal dose and duration of infusion are still unknown.

Central line and congenital heart disease appear to be the most important associated risk factors for thrombosis in neonates and children.^1^ The risk of acute thrombosis in catheterization procedures reaches 10%.^2^ The incidence of clinically apparent thrombosis in neonates (excluding stroke) was 2.4 per 1000 from the Canadian registry and 5.1 per 100 000 symptomatic thromboses as recorded in the German registry.^3^ Arterial thrombosis occurred in 34% and 24% of total thrombotic events in the Canadian and German Registry, respectively. Although the incidence is relatively low, its associated complications can be serious and fatal. Mortality of thrombotic events was 16% in children due to pulmonary embolism and deep vein thrombosis.^3^ Associated morbidity was also high; 8.1% of patients had recurrent thrombosis and 12.4% had post-thrombotic syndrome.^3^

Alteplase is a recombinant tissue plasminogen activator, which initiates fibrinolysis by binding to fibrin in a thrombus surface and converts entrapped plasminogen to plasmin. Its specificity and short half-life are two advantages over the other available thrombolytics. In contrast, the antigenicity and low specificity of streptokinase limited its use in the management of thrombosis in pediatric patients. Urokinase was widely used in pediatric patients, but a US Food and Drug Administration warning has substantially diminished the use of urokinase in North America.^4^ The efficacy and safety of alteplase in adults for management of thromboembolic events is well documented. Recent dose recommendations for alteplase thrombolysis in pediartrics were defined according to site of thrombus and age of the patient.^5^ The available data for the management of thrombosis in neonates and children are predominantly from published case reports and no clear guidelines regarding alteplase use are available yet.^6^–^16^ Therefore, we sought to retrospectively evaluate alteplase efficacy and safety in the management of intracardiac and major cardiac vessel thrombosis in pediatric patients.

## METHODS

In this retrospective case series we evaluated neonates and children patients who developed intracardiac or major cardiac vessel thrombosis and in whom alteplase was used in the cardiac surgery intensive care unit (ICU) at King Faisal Specialist Hospital and Research Center, a tertiary care institution. All neonates and children, 14 years of age or younger, who used alteplase for the management of intracardiac or major cardiac vessel thrombosis from 1997 to 2004, were reviewed. The institutional Clinical Research Committee, as well as the Research Ethics Committee approved the study.

All patients who were less than 14 years old and received alteplase were identified through the pharmacy database at our institution. These patients were sorted by indication and only patients who used the medication for the treatment of intracardiac thrombus or major cardiac vessels were included in the study. Therefore, cases of pulmonary artery, femoral, descending aorta, or portal vein thrombosis and those diagnosed as endocarditis cases were excluded. Furthermore, patients had to have documented cardiac echocardiography done prior to and following alteplase administration. Patients also had to have symptoms of hemodynamic instability or persistent sepsis secondary to the presence of the clot indicating the need for thrombolysis.

Baseline characteristics including patient age, weight, gender, previous medical history, ICU admission period, use of a central venous catheter, total parenteral nutrition administration and whether surgery was done or not before the diagnosis of thrombus, were collected. A pediatric echocardiography consultant reviewed the initial echocardiogram and the baseline thrombus description, location, size, age (echogenic clot indicates an old clot whereas a non-echogenic clot indicates a fresh clot), and determined the degree of blood flow obstruction. Baseline coagulation profile, complete blood count, renal and hepatic profiles were also reviewed. The alteplase regimen including doses given, type of infusion (intermittent or continuous), duration and concurrent medications used were collected.

Post-therapy echocardiograms were reviewed for efficacy of alteplase therapy. Thrombus size and the presence or absence of blood flow and final assessment of dissolution at the time of alteplase discontinuation were assessed. A single echocardiogram specialist analyzed all images. At the time of evaluation, the echocardiographer was blinded to the timing of alteplase administration. Thrombus resolution was categorized as complete, partial, or negligible. Complete clot resolution was defined as the absence of clot as demonstrated echocardiographically on the last day of infusion. The clot was considered partially resolved if its size reduced or blood flow improved from baseline.^17^ Fibrinogen and D-dimer levels used to monitor the response to alteplase were also collected when available.

Baseline and daily blood work collected during and 48 hours after the infusion included hemoglobin, hematocrit, activated partial thromboplastin time (aPTT), prothrombin time (PT), platelets, alanine aminotransferase (ALT), urea, and creatinine. Bleeding complications of the therapy were categorized into three categories: minor bleedings, major bleeding, and death. Bleeding at vascular puncture sites, oozing, or mucosal bleeding were considered minor events if no further sequelae were reported. Major bleeding events were those requiring blood product transfusion as a result of a hemoglobin level decrease of >4 g/dL after therapy, or gastrointestinal, or intracranial hemorrhage or bleeding from more than three sites after infusion.^18^,^19^

## RESULTS

Nineteen pediatric patients were administered alteplase from 1997 to 2004. Six patients were given alteplase for the treatment of either intracardiac or major cardiac vessels thrombus, but only five had completed echocardiographic evaluation and thus met the study selection criteria. The sixth patient was treated with a single alteplase infusion, but he died shortly after and no further echocardiogram evaluation was done, and therefore he was excluded.

The baseline characteristics of the five patients are summarized in Table 1. Patient ages ranged from 40 days to 13 years and three were male. Only one case (Case B) had a genetic related homeopathy (Bahcet disease). A central venous catheter was placed before starting alteplase therapy in four patients (80%), for a median duration of 16 days (1 day up to 30 days). All five patients had compromised hemodynamics and four developed sepsis during their ICU stay. Three patients were started on total parenteral nutrition for various periods. However, none of the study patients had any malignancy.

**Table 1:** Baseline patient's characteristics.

Case	Age	Gender	Weight (kg)	ICU stay^a^ (days)	Central venous catheter^a^	Patient history	Surgery	Sepsis
A	9 y	M	17	0	N	CHD, acute renal failure Bahcet disease, superior sagittal sinus	Fontan	Y
B	13 y	M	46	0	Y	Thrombosis, pulmonary embolism	Tricuspid valve replacement	N
C	40 d	F	3	42	Y	CHD (PDA, abnormal coronary arteries)	Arterial switch	Y
D	7 m	F	3.5	0	Y	Metabolic disease, chronic diarrhea of unknown cause, TPN dependent, impaired coagulation	None	Y
E	65 d	M	1.2	54	Y	CHD, VSD repair, PDA ligation	VSD and ASD closure	Y

a Before alteplase treatment, ASD: atrial septal defect, CHD: congenital heart disease, F: Female, M: Male, m: month; d: day, y: year, PDA: patent ductus arteriosis, TPN: total parenteral nutrition, VSD: ventricular septal defect

One patient was on anticoagulation therapy (warfarin in a dose of 2.5 mg daily) that was stopped upon commencing alteplase treatment (Case A), and another patient was receiving aspirin therapy (Case D). None of the five patients had a history of use of alteplase or any other thrombolytic. All patients received concomitant prophylactic heparin infusion according to the cardiovascular surgery intensive care unit protocol at our institution to achieve a target aPTT of 60-80 sec. The alteplase regimens are summarized in Table 2. The initial bolus of alteplase ranged from 0.3 mg/kg to 0.6 mg/kg followed by an infusion that ranged between 0.05 to 0.5 mg/kg/hr. Alteplase infusion time ranged from 2 hours to 96 hours.

**Table 2:** Alteplase dosing regimens and concomitant antithrombotic agents for the five patients.

Case	Initial dose (mg/kg)	Type of infusion	Alteplase protocol	Total infusion time
A	0.5 (over 6h)	Intermittent	0.5 mg/kg/hr (6 hrs)	19 hrs
B	None	Intermittent	0.5 mg/kg/hr (6 hrs)	2 hrs
C	0.3 (over 6h)	Continuous	0.1 mg/kg/hr, decreased to 0.05 mg/kg/hr, then increased to 0.1 mg/kg/hr	96 hrs
D	0.5 (over 1h)	Continuous	0.2 mg/kg/hr, increased to 0.5 mg/kg/hr, then decreased to 0.2 mg/kg/hr, then increased to 0.5 mg/kg/hr	72 hrs
E	0.6 (over 1h)	Continuous	0.2 mg/kg/hr×4 hr, increased to 0.35 mg/kg/hr, then to 0.5 mg/kg/hr	48 hrs

The results of the echocardiography studies, efficacy and safety of the therapy in the identified five patients are summarized in Table 3. At baseline, echocardiography studies revealed total blood flow obstruction by the clot in two cases, moderate blood flow obstruction in two cases and no blood flow obstruction in one case. The two patients who received intermittent alteplase infusion and had “fresh” clots (Cases A and B) experienced complete clot lysis. The third patient with “fresh” clot (Case D) had a partial response as clot size was decreased, but the resolution of the blood flow obstruction was incomplete. Failure of treatment “negligible response” was documented in two patients; both had “old clots” at baseline and complete blood flow obstruction (Cases C and E).

**Table 3:** Echocardiographic thrombus description at initial diagnosis and response to alteplase for the five patients.

Case	Site	Age of thrombus	Size (mm)	Severity of obstruction	Thrombus resolution	Safety outcomes
A	Fontan lateral tunnel	Fresh	2.5×3.7	Moderate	Complete	Minor bleeding
			1.7×2.3	Mild		
			None	None		
B	Anterior leaflet of the tricuspid valve	Fresh	1×0.5	None	Complete	Minor bleeding
			0.4×0.1	None		
			None	None		
C^a^	Left internal jugular vein	Old	Total occlusion	Severe	Negligible	Major bleeding
			1×1	Moderate		
D	Superior vena cava	Fresh	0.8×0.9	Moderate	Partial	Major bleeding
			0.8× 0.8	Mild		
E	Superior vena cava	Old	1.5×1.1	Total	Negligible	Major bleeding and death
			1.5×1.1	Subtotal		

a By Doppler ultrasound

All patients experienced bleeding events through different sites. Three patients (60%) had major bleeding events as previously defined (Cases C, D, E). All of the three cases received a continuous infusion of alteplase. Case C bled from three sites (endotracheal tube, chest tube and urinary tract) requiring multiple blood products transfusions. Case E bled profoundly through multiple injection sites (despite topical thrombin applications) and from the chest tube with a drop in hemoglobin of 2.5 g/dL, thus requiring multiple transfusions, and eventually died. Case D had severe melena with a drop of hemoglobin by 4.5 g/dL requiring multiple transfusions as well. The other two patients had minor bleeding events. There was oozing around chest tube and injection site in Case A and hemoptysis in Case B, although they were transfused at least once for reasons other than alteplase-related bleeding (blood products transfusion was commenced according to hemoglobin levels and platelets levels; to maintain hemoglobin level >100 g/dL, and platelets >50×10^3^/mm^3^).

## DISCUSSION

In our case series, the decision to start thrombolytic therapy was individualized with no standard protocol for alteplase use followed at our institution for the pediatric population. A median dose of 0.35 mg/kg/hr with a maximum infusion dose of 0.5 mg/kg/hr was associated with a 60% patency rate (2 complete, 1 partial lysis) achieved in an average duration of three days. The three cases had “fresh” clots; two were receiving intermittent dosing of alteplase and experienced no major bleeding complications. Hence, they required lower dosing, a shorter duration of therapy, and had a better outcome in comparison with the other two patients who had “old clots”. The correlation of the clot age to thrombolysis efficacy needs further study; however, some investigators believe that for thrombolytic therapy to be effective the clot should be <3-5 days old and the presence of an organized clot (beyond 1-3 weeks of estimated age) may not respond to thrombolytic therapy.^20^,^21^ If this is true, then we might need to critically screen our patients to identify those who are best candidates for thrombolytic therapy that would respond with the lowest effective dose and would have the lowest risk of hemorrhagic complications.

The recommendations for antithrombotic therapy including thrombolytics in children have been extrapolated from adult recommendations and are not well supported.^4^,^14^ This is mainly due to the differences in the haemostatic system of children, making fibrinolytic therapy more difficult to manage. As such, there is no standardized dosing regimen for alteplase use in the neonatal population. Gupta and colleagues used a bolus of 0.1 mg/kg over 10 minutes followed by an infusion of 0.3 mg/kg/hr for 8 hours with good efficacy and safety, while Hartman's protocol used an initial bolus of 0.7 mg/kg over 30-60 minutes followed by a continuous infusion of 0.2 mg/kg/hr leading to a 94% patency rate without major bleeding complications.^13^,^14^ Higher doses have been used effectively with no complications, including a case report by Mathur and colleagues where alteplase was used in escalating doses up to 0.25 mg/kg/hr for 6 hours for 9 days leading to the dissolution of a large organized intra-atrial cathether tip thrombus.^22^ The doses of alteplase used in our case series are within the dose range used in previously reported cases in the management of pediatric thrombosis (0.01-1 mg/kg/hr with or without bolus dose).^6^–^16^ Moreover, as in our cases, the outcome in these cases varied from no lysis of the clot to partial or complete resolution. When encountered, bleeding ranged from minor as from puncture sites to major intracranial hemorrhage. Two of our patients had minor bleeding, while three had major bleeding complications. Both patients who developed major bleeding received alteplase for a prolonged period of time (48-72 hours). Previous cases of the use of alteplase in the neonates and children have used alteplase for up to 240 hours, but most used alteplase for less than 24 hours.^6^–^16^ The incidence of bleeding in these cases varied from minor to major (up to 56%), but no definitive correlation between the duration of the infusion and the incidence of hemorrhagic complications can be concluded. Furthermore, two of the major bleeding cases were in neonates and one was a preterm. Previous reports have suggested that preterm patients, due to their prematurity, are at an increased risk of bleeding.^23^ This could be another reason for these patients to receive lower doses and a shorter duration of therapy; however, further studies are needed to confirm this finding.

In our series, fibrinogen levels were documented in four patients after the initiation of alteplase and we could not identify a direct correlation between these levels and the time of infusion; however, all of the results were above the recommended levels. It is well known that the fibrinogen level and its degradation products are useful safety and efficacy monitoring parameters, and can guide alteplase infusion adjustment if followed regularly. A fibrinogen level was recommended by Zenz et al, Gupta et al, and required in Levy's protocol.^7^,^10^,^14^ Fibrinogen levels need to be maintained above 100 mg/dL and measured before initiation of alteplase and 4 hours later or when there is dose adjustment. However, adequate monitoring of degradation products and fibrinogen levels does not insure that the patient will not experience bleeding complications. Mueller and colleagues showed that there is no statistically significant correlation between bleeding complications and fibrinogen levels in patients with myocardial infarction who were receiving alteplase.^24^

Our results should be interpreted with caution because of the retrospective nature of the study in addition to the lack of complete laboratory data to evaluate the influence of alteplase on parameters like fibrinogen and D-dimers that can be an excellent monitor of bleeding outcomes. The small sample size and the wide age variation of our subjects are two other limitations of our study.

In conclusion, alteplase in an initial dose of 0.3 up to 0.6 mg/kg along with heparin therapy and followed by continuous, intermittent infusion may effectively dissolve intracardiac thrombi in the pediatric population especially when the thrombus is fresh. A longer duration of therapy was associated with an increased risk of major bleeding. Optimal dose, duration of infusion and clear monitoring guidelines still need to be identified. Further prospective studies with a large sample size evaluating the efficacy and safety of alteplase management of cardiac-related thromboembolic events in pediatric patients are warranted.
